# An evaluation of the evidence brief for policy development process in WHO EVIPNet Europe countries

**DOI:** 10.1186/s12961-022-00852-z

**Published:** 2022-05-07

**Authors:** Adrianna Murphy, Maja Šubelj, Balázs Babarczy, Kristina Köhler, Evelina Chapman, Polonca Truden-Dobrin, Kathryn Oliver, Saskia Nahrgang, Marge Reinap, Tanja Kuchenmüller

**Affiliations:** 1 Department of Health Services Research and Policy, London School of Hygiene and Tropical Medicine, 15-17 Tavistock Place, London, WC1H 9SH United Kingdom; 2 National Institute of Public Health, Trubarjeva 2, 1000 Ljubljana, Slovenia; 3 Faculty of Medicine, University of Ljubljana, Vrazov trg 2, 1000 Ljubljana, Slovenia; 4 National Public Health Center, Albert Flórián út 2-6, Budapest, 1097 Hungary; 5 WHO Country Office in Estonia, Paldiski mnt 81, 10614 Tallinn, Estonia; 6 WHO Regional Office for Europe, Marmorvej 51, 2100 Copenhagen, Denmark

**Keywords:** Evidence-informed policy-making, Knowledge translation, Health research systems, Health policy

## Abstract

**Background:**

Evidence briefs for policy (EBPs) represent a potentially powerful tool for supporting evidence-informed policy-making. Since 2012, WHO Evidence-Informed Policy Network (EVIPNet) Europe has been supporting Member States in developing EBPs. The aim of this study was to evaluate the process of developing EBPs in Estonia, Hungary and Slovenia.

**Methods:**

We used a rapid appraisal approach, combining semi-structured interviews and document review, guided by the Medical Research Council (MRC) process evaluation framework. Interviews were conducted with a total of 20 individuals familiar with the EBP process in the three study countries. Data were analysed thematically, and emerging themes were related back to the MRC framework components (implementation, mechanisms of impact, and context). We also reflected on the appropriateness of this evaluation approach for EVIPNet teams without evaluation research expertise to conduct themselves.

**Results:**

The following themes emerged as important to the EBP development process: how the focus problem is prioritized, who initiates this process, EBP team composition, EBP team leadership, availability of external support in the process, and the culture of policy-making in a country. In particular, the EBP process seemed to be supported by early engagement of the Ministry of Health and other stakeholders as initiators, clear EBP team roles and expectations, including a strong leader, external support to strengthen EBP team capacity and cultural acceptance of the necessity of evidence-informed policy-making. Overall, the evaluation approach was considered feasible by the EBP teams and captured rich qualitative data, but may be limited by the absence of external reviewers and long lag times between the EBP process and the evaluation.

**Conclusions:**

This process occurs in a complex system and must be conceptualized in each country and each EBP project in a way that fits local policy-making culture, priorities, leadership and team styles, roles and available resources. The use of a rapid appraisal approach, combining qualitative interviews and document review, is a feasible method of process evaluation for EVIPNet member countries.

**Supplementary Information:**

The online version contains supplementary material available at 10.1186/s12961-022-00852-z.

## Background

The idea of evidence-informed policy, where policy-making decisions are informed by the latest reliable scientific findings, has been promoted for over two decades [1]. Yet, uncertainty remains about the best approaches to achieving evidence-informed policy-making. Evidence briefs for policy (EBPs) represent one potentially powerful tool for supporting evidence-informed policy-making [2]. An EBP is an evidence synthesis document that involves a systematic approach to contextualizing evidence from systematic reviews, integrating that with context-specific evidence and highlighting the implementation considerations relevant to each evidence-based policy option. The general aim of an EBP is to encourage policy-makers to engage with scientific evidence, develop beliefs that are evidence-informed and propose policy responses that align with this evidence [3].

Previous analyses of policy briefs in general highlight the various factors that can contribute to their effectiveness or to the likelihood of uptake of policy options presented in the briefs [4]. These might include, for example, how clearly and concisely information is presented, the reader’s prior beliefs or their self-perceived level of influence [2, 3], the relationships between researchers producing the briefs and policy-makers [5], or other factors that vary depending on the policy-maker’s role, for example whether the policy brief is story-focused or data-focused [6]. But the *process* of developing the EBP document itself, involving multiple actors and decisions, is likely also affected by various factors. To develop an EBP involves several decisions, including who should lead the process, who else should be involved in the process and what priority area to focus on [7]. Tools to guide the process of developing EBPs exist (for example, WHO’s EBP manual [7] and SURE [Supporting the Use of Research Evidence] guides, [8] and Lavis et al.’s SUPPORT [Supporting Policy Relevant Reviews and Trials] tools [9]). However, to the best of our knowledge, no evaluation has been conducted of the process of developing an EBP. An evaluation of the EBP development process can support its effectiveness by identifying key factors that affect its implementation [10].

### WHO Evidence-Informed Policy Network (EVIPNet) Europe

**Fig. 1:**
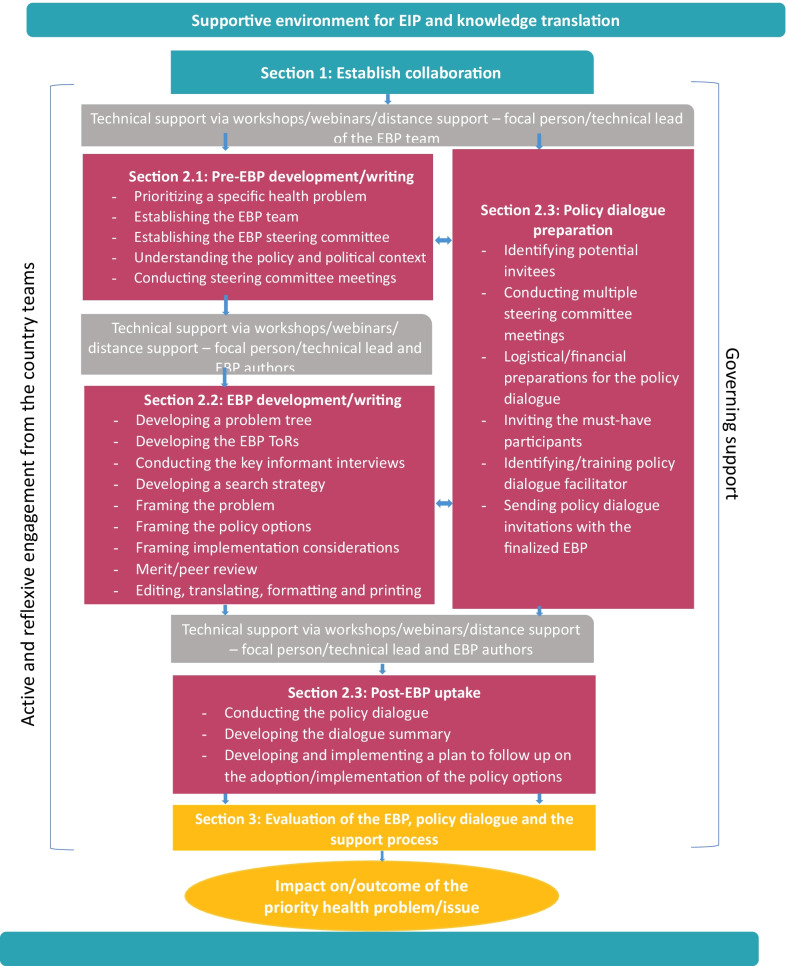
Framework for supporting countries in developing and implementing an integrated knowledge translation approach

In 2005, WHO established the EVIPNet with the goal of minimizing the gap between research and policy-making. EVIPNet Europe was established in October 2012 by the Division of Information, Evidence and Research at the WHO Regional Office for Europe, hosting the WHO Secretariat of EVIPNet Europe, and currently includes 23 Member States. It is supported by the WHO Secretariat of EVIPNet Europe, which is hosted at the Regional Office. EVIPNet Europe aims to promote evidence-informed policy in member countries through a series of activities designed to support the systematic use of health research evidence in policy-making. Among these activities is the process of developing an EBP. The process of developing an EBP is outlined in Fig. 1. It involves steps that take place:

i. prior to EBP development and writing—e.g. prioritizing a problem;
ii. during EBP development and writing—e.g. summarizing the evidence and framing policy options; and
iii. post-EBP development—e.g. encouraging uptake through policy dialogue.

EBPs are written and developed by Member State teams, with technical support in the process from the WHO Secretariat. WHO recommends that EBP team composition is informed by a stakeholder mapping and that teams should include both subject matter experts and members who are familiar with methods for systematically searching and appraising evidence. In practice, the composition and size of EBP teams is influenced by resources available to the Member States and by the roles and relationships of different organizations in the policy-making process in each country [7].

To the best of our knowledge, the process of developing an EBP has not been evaluated. To promote learning and continual improvement of the EBP process, the WHO Secretariat of EVIPNet Europe collaborated with an independent researcher and three EVIPNet Europe country teams (Estonia, Hungary and Slovenia) to evaluate the EBP process in each of these countries.

## Methods

### Study aim

The primary aim of this study was to evaluate the process of developing EBPs in Estonia, Hungary and Slovenia using a rapid appraisal approach. A secondary aim was to assess the feasibility of the evaluation method itself, as an approach that can be applied by EBP team members without evaluation research experience. The results of the study will be used to help EVIPNet country teams identify pitfalls and best practices of EBP development, while also helping them conduct such evaluations themselves. As the focus of the evaluation was on the EBP development *process* rather than on EBP *effectiveness*, the scope of the evaluation included the EBP process up to but excluding post-EBP uptake (see Fig. 1).

### Process evaluation framework

Our study was informed by the United Kingdom Medical Research Council’s (MRC) framework for process evaluations of complex interventions [11]. Multiple process evaluation frameworks exist, with no consensus on a single best approach [11, 12]. We aimed to identify a framework that (i) captured the complexity of the EBP process, which involves multiple interacting components, including the EBP team, expert reviewers, policy-makers and other stakeholders, and the political and social country context; (ii) was broad enough to allow for adaptation to diverse contexts; and (iii) has been validated through use in health-related process evaluations previously. The MRC framework met these criteria. The framework outlines three domains that should be addressed in a process evaluation: (i) implementation (e.g. What is implemented and how?); (ii) mechanisms of impact (e.g. How does the process produce results?); and (iii) context (e.g. How does context affect implementation?).

### Study design: rapid appraisal

The WHO Secretariat of EVIPNet Europe’s aim was to develop an evaluation approach that could be implemented by Member States themselves in future evaluations. As such, consideration was given to practical constraints, including the limited time, financial resources, and social science research skills of the country teams. In the last few decades, approaches that can provide decision-makers with evidence in a timely manner, with minimal resources and without compromising trustworthiness have emerged, so-called rapid evaluation, assessment and appraisal methods [13]. One method increasingly used in time- and resource-limited settings is rapid appraisal (RA) [14]. RA consists of data collection from multiple sources, such as qualitative interviews with stakeholders, secondary data, or document review, to provide an understanding of a situation in a more timely and cost-effective manner than standard social research methods (e.g. surveys). The method is “rapid” in that it is not concerned with achieving a random sample or conducting long-term data collection for statistical precision or generalizability. Rather, it aims to capture a diverse range of perspectives relevant to the specific evaluation context. Triangulation of data from multiple sources provides internal validity and reliability of the data collected. RAs have previously been successfully used for understanding health system processes, including in the WHO European Region [15, 16].

### Data collection and analysis

For this evaluation, we focused on the EBP development process in Estonia, Hungary and Slovenia. These countries had recently completed EBPs (see Box 1) and thus offered an opportunity to provide lessons to other member countries preparing for their own EBP development.

Box 1: WHO EVIPNet Europe EBPs included in process evaluation

- Reducing the consumption of sugar-sweetened beverages and their negative health impact in Estonia (2017)
- Promoting the appropriate use of antibiotics to contain antibiotic resistance in human medicine in Hungary (2018)
- Antibiotic prescribing in long-term care facilities for the elderly in Slovenia (2018)

Data for this study were collected in July and August 2019. In Hungary and Slovenia, data collection was led by the EVIPNet country team leads (BB, MS, respectively). In Estonia, data collection was led by AM, an independent researcher seconded to EVIPNet Europe to support the evaluation. Data were collected using semi-structured interviews and document review. Participants for interviews in each country were purposively selected to capture perspectives of as many people as possible who were involved, directly or indirectly, with the EBP process. These included members of the EBP team, subject matter experts who had been invited as external reviewers of the EBPs, WHO country office representatives, and other local stakeholders familiar with the EBP. The total number of participants was 5 each in Hungary and Estonia and 10 in Slovenia. Interviews were conducted by the lead evaluator and semi-structured to allow for unexpected themes to emerge. In Hungary and Slovenia, interviews were conducted in the local language; in Estonia, interviews were in English. The topic guide for the interviews aimed to address each step in the EBP process, aiming to capture data on the implementation process (what EBP development steps were actually implemented and how), experiences of participants’ interaction with the process, and the contextual factors that affected all of these [11]. The English version of the topic guide is included as Additional file 1: Appendix S1. For the document review, relevant documents included EBP team terms of reference and work plans, published and in-progress EBPs, internal “lessons learned” reports written by EBP teams and other notes from EBP teams.

Data analysis for both interview data and documents followed steps of thematic analysis outlined by Braun and Clarke [17], including initial open coding of data, identification of core codes and constant comparative analysis to develop categories, generation of themes, and reflexivity to ensure against the influence of preconceived assumptions. Emergent themes were related back to the MRC framework to identify factors relating to implementation, mechanisms of impact and context that affected the EBP development process. Finally, after concluding the process evaluation, those who led the evaluations in each country, and other EVIPNet Europe representatives from other countries, gathered at a workshop in September 2019. The aim of the workshop was to reflect on the results, their experiences of the evaluation approach and to identify lessons for those who might conduct evaluations of the EBP development process in the future.

## Results

We identified key themes affecting the process of EBP development within each of the three MRC process evaluation framework components—implementation process, mechanism of impact, and context. Our findings are described below and summarized in Tables 1, 2 and 3 by MRC framework component.

**Table 1:** Themes emerging relating to the EBP development implementation process in EVIPNet Europe member countries

Country	How the process was initiated/problem prioritized	Team composition and roles
Estonia	Piggy-backing on existing government policy priority and topic selected by the Ministry of Social Affairs resulted in increased engagement, resources and attention to the EBP	Roles and responsibilities were defined early in the process and supported efficient collaboration
Hungary	Including only technical staff from the Ministry of Health (vs policy decision-makers) limited political support	Roles and responsibilities not clearly defined from the beginning, hampering early progress
Slovenia	Topic not chosen at high-level of Ministry of Health and this compromised their ownership and endorsement of the process	Roles and responsibilities were defined early in the process and supported efficient collaboration.

**Table 2:** Themes emerging relating to the EBP development mechanisms of impact in EVIPNet Europe member countries

Country	Leadership	External support
Estonia	Effective leadership to maintain motivation and commitment throughout the process	Continued capacity-building and the review of EBP drafts by external expert EVIPNet team (EVIPNet Chile) provided encouragement and peer support
Hungary	Effective leadership of EBP team lead vital to seeing the process through	The WHO Country Office provided legitimacy and political support to the process
Slovenia	Leader who acted as EBP champion was vital for maintaining motivation	Consistent support and input from WHO filled a gap where Ministry of Health engagement was unreliable

**Table 3:** Themes emerging relating to the EBP development context in EVIPNet Europe member countries

Country	Culture of policy-making
Estonia	Political situation (changes in government and associated interests of government) can determine interest in EBP Value of evidence is recognized due to policies mandating the application of research evidence (i.e. impact assessments of all proposed legislation)
Hungary	No established practice of evidence-informed decision-making processes
Slovenia	Awareness of and familiarity with the EBP process among policy-makers will determine their engagement in applying its evidence-informed options

A. Implementation process
  i. How the EBP process was initiated
    In the EBP process, the first step in the actual EBP development is to prioritize a health problem based on local evidence. The assumption is that selecting a problem based on local evidence of the relevance of this problem will increase the likelihood of policy-maker engagement in the EBP process. But *how* the problem was prioritized in each of the three study countries emerged as important to the perceived engagement of policy-makers in the EBP development process. And even before this step, it seemed that who was involved in *initiating* the EBP process—most relevant to the “Establishing the collaboration” step that occurs even before beginning EBP development—also seemed to play an important role in stakeholder engagement. Particularly, in Slovenia and Estonia, whether or not the Ministry of Health (MoH) (or in the case of Estonia, the Ministry of Social Affairs (MoSA), which has responsibility for health) played a leading role in initiating the EBP process or in prioritizing the problem to be focused on in the EBP, appeared to dramatically affect MoH stakeholders’ engagement and support for the EBP process. In Estonia, the EBP team had members from the MoSA and conferred with the MoSA to identify a problem on which to focus the EBP (sugar-sweetened beverages) that had already been agreed by the government as a policy priority. The MoSA had already been tasked with analysing policy options to address this problem. The EBP team thus benefitted from heightened interest on the part of not only the MoSA but other ministries regarding the evidence and policy options that would be highlighted in the EBP and were able to secure meetings and input from these stakeholders. In Slovenia, a “bottom-up” approach was used, where the EBP team, comprised of staff from the National Institute of Public Health (NIJZ), initiated the EBP process with support from WHO and selected the priority problem—antibiotic prescription in long-term care facilities. Antimicrobial resistance is an internationally agreed health priority, and the Slovenian team selected the specific focus on prescribing in long-term care facilities based on local evidence of irrational prescription in these facilities [18]. However, this was not an issue prominently featured in the MoH’s policy agenda at the time nor selected by MoH policy-makers. As demonstrated in the following quotations from the Slovenian data, the fact that the EBP process was not initiated by the MoH itself and that the problem addressed by the EBP was not selected by the MoH may have impeded MoH interest and engagement in the whole EBP process. This may also thus affect the potential long-term impact of the EBP to inform its policy decisions.
    *Maybe the way we handled the whole process it was suboptimal. Because basically, instead of being ordered to do this, we went from the bottom up…then we have to try to convince those who should use this tool to use it already…We were not successful in achieving that they be part of our work and that our work would be translated into decisions by those who would use it….* (male, Slovenia)
    *I anticipated that several stakeholders at the national level and policy-makers would be involved in the process. If the problem does not shift from the theory into practice, then this is a real disadvantage. Therefore, it would be necessary to involve several decision-makers in the process itself.* (female, Slovenia)
    This was important in Hungary as well, where selection of the priority problem for the EBP involved technical staff from the MoH but not staff involved in policy decisions. Thus, MoH engagement was primarily comprised of technical input rather than political support. Taken together, these experiences suggest that to encourage policy-maker engagement throughout the process, it may be important to do more than select a problem that “should” be of interest to them, but instead to engage staff in various MoH functions from the start in initiating the process (i.e. agreeing on the need for an EBP, selecting the EBP team) and determining its direction, including the prioritization of a specific problem for the EBP.
  ii. EBP team composition and roles
    Another emerging theme was related to the composition and roles of the EBP team. The second step in implementing the EBP process is identifying a core EBP team that will be responsible for all aspects relating to the EBP. Guidance for EBP development advises establishing a team that comprises a methodological lead, administrative lead, evidence-synthesis lead, topic experts and external support (i.e. WHO Country Office), although one team member can fill more than one role. However, we found that the composition of teams varied depending on country context (team composition is described in Additional file 1: Appendix S2), and this affected the perspectives that were included in the EBP development and thus potentially the perceived applicability of the EBP to users. For example, in Hungary, the EBP team largely consisted of government officials and university researchers, and only one clinician (from the hospital level). Further clinicians (including general practitioners) and policy-makers were invited to provide comments on the EBP, but were not intimately involved in its development. The result was an EBP that was perceived by some stakeholders as not sufficiently taking practical or policy considerations into account.
    *I don’t know how it could be arranged, but others should have also been involved on the way. It is fine that the three or four [government] experts had this idea about the whole topic. […] But there is public administration which has a view about this set of issues, and maybe this should have been contrasted to a sharp opinion of practical experts [prescribers] from the field, and the final product should be based on both. Because this way, three experts wrote the whole thing. Even if we ask one or two people from the field, they would then offer some comments, but it doesn’t give the same results as if they worked on it the same way [as the team members did]. […] Not like this, that we make a document with only three of us [lead authors] and other people only comment here and there.* (female, Hungary)
    The team’s efficacy seemed to also be affected by the roles and responsibilities of team members and how explicitly these were defined. In Slovenia and Estonia, it was generally felt that roles and responsibilities were clearly and efficiently allocated and maintained. However, in Hungary, an unclear definition of each member’s role and the expected time commitment seemed to result in lost time. Guidance on EBP development suggests that people from different perspectives should be included in the EBP development to support different aspects of EBP development (and eventual uptake). And indeed, our interviews, as above, suggested that more diverse teams, including members from different sectors, would result in more widely accepted or applicable EBPs. But other interviews suggested that in practice, a small core team might be necessary to improve accountability and thus make the process more efficient:
    *I think it’s a lot more difficult to work in a team than one would expect […] there certainly was an uneven distribution of the workload. Probably when the joint work started, with a headcount of around 10, then it was not really clear how many working hours each one was able and willing to commit to actually writing the brief. […] Maybe we could have addressed this type of challenge more easily if at the beginning, if consideration had been given to this issue, or if someone had drawn attention to this, maybe on behalf of WHO, that effectively, there have to be one or two—maximum three—people who, as lead authors, steer the development of the brief.* (female, Hungary)
    This suggests that to better reflect the views of various stakeholders, EBP teams should at least be large enough to include diversity in terms of areas of expertise, but at the same time, to encourage accountability, there should be a small number of specific individuals with explicit responsibility for EBP delivery.
B. Mechanisms of impact
  i. Leadership
    The EBP team leader should normally be the first person to be recruited and, according to guidance, is the focal person and method expert. In practice, they may be recruited at the same time as the rest of the team and may not have previous experience in leading a team or EBP development. Regardless of their area of expertise and past experience, however, the leadership ability of the EBP team leader seemed to be valued as important to the perceived success of the development process. In all three countries, effective team leadership contributed to maintaining motivation among team members, as the leader took on the responsibility of making key decisions, steering the working group and championing the cause of the EBP to policy-makers and other stakeholders.
    *I can still recall how persuasively [EBP lead author] could explain that at last, we could do something. […] If it wasn’t for her, I may have dropped out of this [project].* (female, Hungary)
    *The team leader was very organized. She assigned concrete responsibilities and timelines to each team member and as well as the regular capacity-building webinars helped to keep track.* (female, Estonia)
  ii. External support
    Support from an external partner also emerged as important to sustaining the EBP process, particularly in contexts where political support was unreliable. In Slovenia, perhaps particularly due to the limited engagement of the MoH as described above, the WHO EVIPNet Europe Secretariat played a crucial supporting role by providing not only technical support on EBP development but also by lending its name and credibility to the process, for example in communications with stakeholders, as well as providing financial support.
    *Based on these years of experience, we see, that if we participate in such a process under the auspices of the WHO, this cooperation protects such a process, and increases the chances of the EBP process being successful. Because when we look at our political situation, we are very vulnerable because we have many changes in the ministry. Due to changes in the ministry, priorities are then changed, and people get other tasks at the NIJZ, which in turn complicates the successful implementation of the EBP process and its continuity.* (female, Slovenia)
    In Estonia, support came from peers in EVIPNet America who had significant previous experience in developing EBPs and in EBP-related guidelines.
    *The support we got from EVIPNet Chile was important for us. They gave us some guidance in conducting literature searches properly and provided online trainings. This helped with motivation because it reassured us, we were on the right track.* (female, Estonia)
C. Context
  i. National culture of policy-making
    Our interviews also elucidated the impact that contextual factors can have on the process’ direction and likely outcomes. In particular, the national culture of policy-making emerged as a fundamental contextual factor in the engagement of policy-makers in the EBP process (and thus its likely impact). What was specifically most cited is the degree to which the processes of evidence-informed policy-making were entrenched in the country or understood and accepted by policy-makers, compared to policy-making driven by self-interest or other political forces. In Slovenia and Hungary, the fact that evidence-informed policy-making processes like EBP are unfamiliar and not yet institutionalized was used to explain the lack of commitment of policy-makers to these processes.
    *My impression is that decision-makers do not know the process precisely and, therefore, have not been actively involved in it. […] Of course, this is in line with the broader social situation in the country. […] We have no established evidence-based decision-making paths. There is no active opposition to this process. It is merely that decision-makers or those who are the stakeholders do not recognize this as something that can help them in decision-making.* (male, Slovenia)
    *There are areas where evidence does inform policy in Hungary, e.g. hypertension and rheumatoid arthritis treatment guidelines and financing protocols—but antibiotic use is not among these. Maybe a policy broker would be needed to support knowledge translation in this area.* (male, Hungary)
    On the contrary, in Estonia, there was an overall sense that the culture of policy-making was becoming increasingly conducive to evidence-informed policy-making processes. This included legislative elements that support evidence-informed policy-making overall, including a compulsory impact assessment for all new legislative policy proposals.
    *It feels as though more and more they (policy-makers) see that evidence is essential for policy-making, and it helps that there are now these rules in place that support something like the EBP as part of the official process, not just something that a small number of people think is important. We have now a law that new policies have to go through a rigorous impact assessment, and the EBP can contribute to this. There are also now opportunities for public input into the development of new policies.* (female, Estonia)
    Despite the fact that the EBP process was unfamiliar in Hungary, there was optimism that this process—by demonstrating that experts from different backgrounds could work together to produce meaningful policy recommendations grounded in evidence—might raise awareness of the potential of EBPs and make an incremental contribution to changing the culture of policy-making in the country.
    *I really enjoyed that this process took place, that this whole project took shape. That we were able to explain a slice of this problem. […] that we could work together in this, I think this could address a very deep gap. […] We are working in a field where you’ll never have the Nobel Prize, and we cannot discover new things, but the very beauty of it is that it can alter everyday practice, if this [information] is channelled to those who can make a change.* (female, Hungary)
    In addition to our evaluation results, the research team’s reflections on the application of the evaluation approach itself provided useful lessons for future EBP process evaluations. Overall, the approach was considered feasible, and the use of qualitative methods was deemed advantageous for exploring nuances in terms of the experiences of those involved in the EBP process. While the inclusion of those most familiar with the EBP process as interview participants was considered very important for capturing detailed data on the factors affecting this process, there was uncertainty as to whether those involved in the process could maintain objectivity as process evaluators. It was also felt that the sooner the evaluation could occur after completion of the EBP process, the more fruitful such an evaluation would be. Finally, while document review was helpful for familiarizing the one external evaluator with the EBP process in each country, it was the qualitative interviews that offered richer data on factors affecting these processes. Reflections shared from each country are detailed in Box 2.

Box 2: Reflections on the approach used for evaluating EBP processes in Estonia, Hungary and Slovenia Estonia

- Long time lag between EBP process and evaluation may compromise the evaluation as details are forgotten by some.
- Those “leading” or “responsible” for the process appear to have sharpest memory of process and deepest insights.
- Including individuals not directly involved in EBP process may have led to a different (perhaps more critical?) perspective.
- External evaluator may help in achieving objectivity of evaluation.
- Topic guide should really be a “loose” guide, as some participants may lead the discussion in various directions, and their perspective should be allowed to emerge.

Hungary

- Evaluation would be more fruitful and effective if it was done earlier.
- The topic guide is comprehensive and general enough to capture the key issues related to the EBP process.
- The questions on mechanisms of impact and context seem to provide the most valuable and interesting insights.
- The added value of reviewing documents is questionable where documents are very descriptive.
- The remaining objective is challenging given the evaluators’ central role in the EBP process.

Slovenia

- Qualitative data collection allows for exploring experiences, feelings and attitudes, and thus uncovered issues and concerns not anticipated or considered by the researchers.
- Qualitative approach is appropriate for exploring EBP process in Slovenia, where policy-making is often based on perspectives and intuition rather than measurable factors.
- Providing insights into the challenges and successes of the EBP process may motivate stakeholders to become more engaged in the process in the future.

## Discussion

Our research provides valuable insights into factors affecting the EBP development process. Rather than there being a one-size-fits-all approach to EBP development, this process is influenced by, and must take account of, variations in how the EBP processes are initiated, how priority problems are identified, the diversity and size of EBP development teams, what external support and leadership capacity is available, and the national culture of policy-making in a given context. While our findings pertain to the EBP development process specifically, they align with a growing understanding of the whole evidence-informed policy-making process as a complex system [19]. Specifically, it is now increasingly accepted that a linear conceptualization of knowledge translation is insufficient to explain or to ensure the effectiveness of policy-making processes [19, 20]. A linear model suggests there is a deficit in knowledge among policy-makers and that by simply filling that deficit, evidence-informed policies will be adopted. The insufficiency of this model was evident in our findings regarding the influence of how, and by whom, the EBP focal problem was prioritized. For example, while in both Slovenia and Estonia, the EBP focused on problems for which there was evidence of local relevance, and the EBPs produced evidence-based policy options to address this problem, the fact that the MoH in Slovenia was not involved in the initiation of the EBP process or prioritization of the problem impeded their engagement and interest in the EBP process. While we did not evaluate outcomes of the EBP process in this study, we might expect that lower engagement and interest in the EBP process might negatively impact the likelihood of uptake of the policies outlined in the EBP.

Involving the MoH (or other relevant ministry) and other stakeholders in the problem prioritization is recommended by EVIPNet guidance as a means of increasing the likelihood of EBP uptake [7]. And again, while EBP uptake itself was not within the scope of this evaluation, indeed, many potential benefits of engaging stakeholders in policy development overall have been proposed, including identifying and rectifying disagreements, aligning recommendations with societal needs and expectations, and increasing transparency and trustworthiness of the policy development process [10, 21]. But is simply engaging various stakeholder groups like the MoH in the EBP process to jointly prioritize a problem and coproduce policy options enough to improve the EBP process implementation and likely impact? A *relational* model of knowledge translation would suggest so. This is a model that incorporates the linear model discussed above, but builds on it to highlight the importance of “sharing of knowledge, the development of partnerships, and the fostering of networks of stakeholders with common interests.” [19, 22, 23]. Indeed, there is evidence to support the importance of building interest in research evidence among policy-makers and trust between evidence producers and users, and the interactive process required to do so [24–26]. However, as outlined by Best and Holmes [19], there are specific contextual characteristics that must be in place to allow a relational model to be sufficient for understanding policy-making processes. These include contexts where (i) there is consensus about the value and place of evidence-informed policies; (ii) the organizational culture and resource allocation favours evidence-informed policy-making; (iii) the problem being addressed requires a change in the system to support practitioner change, and this is accepted by opinion leaders and decisions-makers; and (iv) the research agenda, structure and resources are stable and support communication and collaboration between researchers and policy-makers. As we observed in our study, these conditions may not be met in all of the countries included. In all countries, the culture of policy-making emerged as a crucial influence on the EBP process. Evidence-informed policy-making processes are not yet fully institutionalized in any of the three study countries. As such, the resources and attention devoted to these processes do not appear sufficient for supporting the EBP teams and process. In Estonia, our interviews suggested stronger support and resources for evidence-informed policy-making processes in the country. However, a sense also emerged that while the EBP was aligned with policy-maker priorities in this instance, and that the accepted practice of using evidence in policy-making supported the need for EBPs, that a change in government could impact the level of support for the EBP or implementation of its recommended policy options.

In these contexts, a more useful conceptualization of evidence-informed policy-making processes is likely a complex systems model. This recognizes that policy-making contexts are made up of multiple agents with unique worldviews, whose interaction is mediated by structures that shape the relationships of these agents and the diffusion and dissemination processes in which they participate, and that these are all part of one system that needs to be “activated” and moving towards a common goal of evidence-informed processes to work. Previous evaluations of evidence translation initiatives have suggested that to work in a complex system, such initiatives must (i) “act scientifically and pragmatically”, with interventions reflecting the unique characteristics of a system and adapting as the system responds; (ii) “embrace complexity”, identifying and addressing the parts of the system that are not functional and will impede an intervention’s implementation; and (iii) “engage and empower”, securing commitment and insights from a range of system stakeholders and aligning interventions with their motivations and concerns [27]. In a complex system, an EBP is just one part of a larger machine, all of which has to be moving towards policy change for the EBP to take hold. In this context, and as we find in our study, EBPs and the process to develop them must be “demand-driven”, that is, driven by the demand from stakeholders and a system already moving towards policy change, rather than imposed. And, as we also found, they must be conceptualized in a way that fits local policy-making culture, local priorities, leadership and team styles, roles and available resources.

### Reflections on the evaluation approach and limitations

Our findings regarding the appropriateness of our methodological approach to this evaluation (i.e. using the MRC process evaluation framework, RA including qualitative interviews and EBP team members as evaluators) supports the use of this approach in other EVIPNet Member States as part of efforts for continual learning and feedback. We find that the approach is feasible and can provide useful insights but is not without limitations. We highlight these limitations and recommendations for EVIPNet country members considering evaluations of EBP processes in the future, as well as other teams interested in evaluation evidence-informed policy-making processes. For practical reasons, and to enable EVIPNet country teams to conduct their own evaluations, in Hungary and Slovenia, the evaluations were led by members of the EBP team. As discussed in the MRC Process Evaluation guidance, in choosing evaluators, it is important to balance close and positive working relationships that enable close observation of the process under evaluation, with the need to ensure credibility of evaluators. In our case, the closeness of the evaluators to the process likely allowed us to capture more detailed data, but we acknowledge that evaluators who were also involved in the process will have inherent biases. To address this, we identified and reflected on these potential biases throughout the evaluation process. The use of one external evaluator in reviewing and synthesizing the findings from each country also helped towards reducing bias. Future EBP evaluations should consider the inclusion of an external evaluator where resources allow. Furthermore, MRC guidance also recommends that evaluation teams have the appropriate skills to apply the evaluation methods. While in our case, guidance and support in qualitative interviews were provided to those conducting the evaluations, they did not all have previous experience with qualitative interviews, and it is possible that methodological support may not be available in all contexts. It would be important to consider options for supporting or strengthening the capacity of those country teams planning similar evaluations.

### Conclusion

The EBP development process is affected by how the focus problem is prioritized, and who initiates this process, by EBP team composition, EBP team leadership, the availability of external support in the process, and the culture of policy-making in a country. This process occurs in a complex system and must be conceptualized in each country in a way that fits local policy-making culture, priorities, leadership and team styles, roles and available resources. The use of an RA approach, combining qualitative interviews and document review, is a feasible method of process evaluation for EVIPNet member countries and other teams interested in evaluating evidence-informed policy-making processes.

## Supplementary Information

**Additional file 1: Appendix S1.** Data collection tool for semi-structured interviews. **Appendix S2.** Composition of EBP teams in each study country.

## Data Availability

Due to the small number of participants the data are likely to be identifiable, and thus we cannot make the qualitative interview data publicly available.

## Abbreviations

EBP: Evidence brief for policy
EVIPNet: Evidence-Informed Policy Network
MoH: Ministry of Health
MoSA: Ministry of Social Affairs
MRC: Medical Research Council (United Kingdom)
NIJZ: National Institute of Public Health (Slovenia)
RA: Rapid appraisal

## References

[1] Walt G (1994). How far does research influence policy?. Eur J Public Health.

[2] Dodson EA, Eyler AA, Chalifour S, Wintrode CG (2012). A review of obesity-themed policy briefs. Am J Prev Med.

[3] Beynon P, Chapoy C, Gaarder M, Masset E. What difference does a policy brief make? Full report of an IDS, 3ie, Norad study. Brighton: Institute of Development Studies; 2012.

[4] Dodd M, Ivers R, Zwi AB, Rahman A, Jagnoor J (2019). Investigating the process of evidence-informed health policymaking in Bangladesh: a systematic review. Health Policy Plan.

[5] Shroff Z, Aulakh B, Gilson L, Agyepong IA, El-Jardali F, Ghaffar A (2015). Incorporating research evidence into decision-making processes: researcher and decision-maker perceptions from five low- and middle-income countries. Health Res Policy Syst.

[6] Brownson RC, Dodson EA, Stamatakis KA, Casey CM, Elliott MB, Luke DA (2011). Communicating evidence-based information on cancer prevention to state-level policy makers. J Natl Cancer Inst.

[7] EVIPNet Europe (2019). Evidence briefs for policy. Using the integrated knowledge translation approach: a guiding manual.

[8] World Health Organization (2011). SURE guides for preparing and using evidence-based policy briefs.

[9] Lavis JN, Oxman AD, Lewin S, Fretheim A (2009). SUPPORT tools for evidence-informed health policymaking (STP). Health Res Policy Syst.

[10] Lavis JN, Lomas J, Hamid M, Sewankambo NK (2006). Assessing country-level efforts to link research to action. Bull World Health Organ.

[11] Moore GF, Audrey S, Barker M, Bond L, Bonell C, Hardeman W (2015). Process evaluation of complex interventions: Medical Research Council guidance. BMJ.

[12] Grant A, Treweek S, Dreischulte T, Foy R, Guthrie B (2013). Process evaluations for cluster-randomised trials of complex interventions: a proposed framework for design and reporting. Trials.

[13] McNall M, Foster-Fishman PG (2007). Methods of rapid evaluation, assessment, and appraisal. Am J Eval.

[14] Beran D, Yudkin JS, de Courten M (2006). Assessing health systems for type 1 diabetes in sub-Saharan Africa: developing a ‘rapid assessment protocol for insulin access’. BMC Health Serv Res.

[15] Murphy A, Chikovani I, Uchaneishvili M, Makhashvili N, Roberts B (2018). Barriers to mental health care utilization among internally displaced persons in the republic of Georgia: a rapid appraisal study. BMC Health Serv Res.

[16] Balabanova D, McKee M, Koroleva N, Chikovani I, Goguadze K, Kobaladze T (2009). Navigating the health system: diabetes care in Georgia. Health Policy Plan.

[17] Braun V, Clarke V (2006). Using thematic analysis in psychology. Qual Res Psychol.

[18] Stepan D, Usaj L, Petek Ster M, Smolinger Galun M, Smole H, Beovic B (2018). Antimicrobial prescribing in long-term care facilities: a nationwide point-prevalence study, Slovenia, 2016. Euro Surveill.

[19] Best A, Holmes B (2010). Systems thinking, knowledge and action: towards better models and methods. Evid Policy.

[20] Simis MJ, Madden H, Cacciatore MA, Yeo SK (2016). The lure of rationality: why does the deficit model persist in science communication?. Public Underst Sci.

[21] Lemke AA, Harris-Wai JN (2015). Stakeholder engagement in policy development: challenges and opportunities for human genomics. Genet Med.

[22] Graham ID, Logan J, Harrison MB, Straus SE, Tetroe J, Caswell W (2006). Lost in knowledge translation: time for a map?. J Contin Educ Health.

[23] Lomas J (2007). Decision support: a new approach to making the best healthcare management and policy choices. Healthc Q.

[24] Ezenwaka U, Mbachu C, Etiaba E, Uzochukwu B, Onwujekwe O (2020). Integrating evidence from research into decision-making for controlling endemic tropical diseases in South East Nigeria: perceptions of producers and users of evidence on barriers and solutions. Health Res Policy Syst.

[25] Ellen ME, Leon G, Bouchard G, Ouimet M, Grimshaw JM, Lavis JN (2014). Barriers, facilitators and views about next steps to implementing supports for evidence-informed decision-making in health systems: a qualitative study. Implement Sci.

[26] Dobbins M, Robeson P, Ciliska D, Hanna S, Cameron R, O'Mara L (2009). A description of a knowledge broker role implemented as part of a randomized controlled trial evaluating three knowledge translation strategies. Implement Sci.

[27] Reed JE, Howe C, Doyle C, Bell D (2018). Simple rules for evidence translation in complex systems: a qualitative study. BMC Med.

