# Supplementary material for: An evaluation of the evidence brief for policy development process in WHO EVIPNet Europe countries
Source: Health Res Policy Syst. 2022 May 7;20:54. doi: 10.1186/s12961-022-00852-z (PMC9077836; doi:10.1186/s12961-022-00852-z)
Supplement: Supplementary file 1 — Additional file 1: Appendix S1. Data collection tool for semi-structured interviews. Appendix S2. Composition of EBP teams in each study country. [file 12961_2022_852_MOESM1_ESM.docx]

Appendix S1: Data collection tool for semi-structured interviews

**SEMI-STRUCTURED INTERVIEW GUIDE**

**Please fill out the following:**

**Location __________________________________**

**Position of respondent (eg EBP team member) __________________________________**

**Respondent's specialty (medicine, policy…) __________________________________**

*Instruction:*

*This is a guide outlining the areas we want to understand. Not all participants will be informed about all these areas or will have the time to answer. Below are some leading questions and examples of follow-up probes but participants may follow their own line of thinking and may not like to be interrupted. They should be given opportunity to talk about issues where they have most expertise and personal interest, with some directing from the interviewer. Before each interview, think about what the role of the participant is and in what areas they can provide perspective.*

**Introduction:**

*Interviewer:* Hello my name is _____________. Thank you very much for agreeing to participate in this interview today. As you have been told, I will be keeping notes of our interview. You are also free to stop the interview at any time or refuse to answer any questions.

1. Could you tell us about yourself and your organization and its relationship to the process of developing an EBP in your country?

*Implementation*

With the following questions I would like to ask you about your perspective on the EBP process:

Pre-EBP planning

2. Can you tell me about your perspective on how the problem was identified? (e.g. Who was involved? How was the problem identified? Were there any facilitators or challenges?)

3. Who was part of the EBP team and how were they identified? What about the steering committee? Who were other stakeholders involved and how were they identified?

EBP writing

4. How were key informants selected for interviews and were these helpful?

5. Can you tell me about how evidence was identified, reviewed and selected? (e.g. Who was involved, how was the relevant evidence identified, reviewed, selected? Were there any facilitators or challenges?)

6. Can you tell me about how the problem was framed? Were there any specific challenges framing the problem in your country? Do you think that the way in which the problem was framed is useful?

7. Can you tell me your perspective on how evidence was adapted to your country context? (e.g. How was local implementation of global evidence considered? Did the EBP accurately reflect contextual factors in your country?)

Post EBP

8. If you participated in the planning of the policy dialogue, can you tell me your perspective on how this went? Were the intended outcomes achieved? Were there any unforeseen challenges?

9.If you participated in the policy dialogue as a stakeholder, can you tell me your views on this event? Did you find it useful?

10. Are you aware of what other outputs of the EBP process? What are your thoughts on the quality and usefulness of these outputs?

11. What were the outcomes of the EBP process? For example, has the EBP any impact on policy or policy-making behavior? Do you think the EBP process achieved its goals? How did it do so?

12. Can you share any thoughts on how the overall EBP process reflected the intended process?

*Mechanisms of impact*

13. How did EBP team members interact with the EBP process? What about reviewers? Policy-makers?

14. Can you tell me about any support you are aware of that was provided to the EBP process and how well this worked?

15. In your view did the process of developing an EBP in your country take any unexpected pathways?

16. What about any unexpected consequences of the EBP process?

*Context*

17. Can you tell me about any political, social, cultural or other factors specific to your country context that affected the EBP process?

18. Can you tell me about any factors external to your country context that might have affected the EBP process in your country?

19. Would any of these factors you’ve mentioned affect how you approach the EBP process in the future?

20. Has the EBP process itself affected the context of evidence-informed policy making in your country? (e.g. Has the EBP process affected attitudes regarding EIP, or attitudes regarding the problem that your EBP addressed?)

*Overall*

21. Is there anything else you would like to tell me about the EBP process? For example, any lessons you learned or things you would do differently in the future?

Appendix S2: Composition of EBP teams in each study country

|  | Hungary | Slovenia | Estonia |
| --- | --- | --- | --- |
| EBP Team | - 2 Health policy analysts - 1 Medical guideline development expert - 3 Epidemiologists - 1 Infectious disease specialist (researcher) - 2 Pharmacologists | - 2 Public health specialists - 3 Infectious disease specialists (clinicians and researchers) | - 1 Policy analyst - 1 Nutrition specialist - 3 Public health specialists - 1 Public health and nutrition specialist |
